# Timing of surgical antibiotic prophylaxis administration: Complexities of analysis

**DOI:** 10.1186/1471-2288-9-43

**Published:** 2009-06-23

**Authors:** Carrie Cartmill, Lorelei Lingard, Glenn Regehr, Sherry Espin, John Bohnen, Ross Baker, Lorne Rotstein

**Affiliations:** 1 The Learning Institute, The Hospital for Sick Children, Toronto, Canada; 2 Department of Pediatrics, University of Toronto, Toronto, Canada; 3 Wilson Centre for Research in Education, University Health Network, Toronto, Canada; 4 Faculty of Medicine, University of Toronto, Toronto, Canada; 5 Daphne Cockwell School of Nursing, Ryerson University, Toronto, Canada; 6 Department of Surgery, St. Michael's Hospital, Toronto, Canada; 7 Health Policy, Management and Evaluation, University of Toronto, Toronto, Canada; 8 Division of General Surgery, University Health Network, Toronto, Canada

## Abstract

**Background:**

The timing of prophylactic antibiotic administration is a patient safety outcome that is recurrently tracked and reported. The interpretation of these data has important implications for patient safety practices. However, diverse data collection methods and approaches to analysis impede knowledge building in this field. This paper makes explicit several challenges to quantifying the timing of prophylactic antibiotics that we encountered during a recent study and offers a suggested protocol for resolving these challenges.

**Challenges:**

Two clear challenges manifested during the data extraction process: the actual classification of antibiotic timing, and the additional complication of multiple antibiotic regimens with different timing classifications in a single case. A formalized protocol was developed for dealing with incomplete, ambiguous and unclear documentation. A hierarchical coding system was implemented for managing cases with multiple antibiotic regimens.

**Interpretation:**

Researchers who are tracking prophylactic antibiotic timing as an outcome measure should be aware that documentation of antibiotic timing in the patient chart is frequently incomplete and unclear, and these inconsistencies should be accounted for in analyses. We have developed a systematic method for dealing with specific problematic patterns encountered in the data. We propose that the general adoption of a systematic approach to analysis of this type of data will allow for cross-study comparisons and ensure that interpretation of results is on the basis of timing practices rather than documentation practices.

## Introduction

The timing of prophylactic antibiotic administration is a patient safety outcome that is recurrently tracked and reported. Compliance to antibiotic prophylaxis guidelines [1-9], the effect of interventions on antibiotic prophylaxis timing [10-20], and the relationship between prophylaxis timing and the incidence of surgical site infection [21-24] are quality measures that are described in the literature. The results of the published work are unanimous in suggesting an association between properly timed antibiotic prophylaxis and a decreased rate of surgical site infections [21-30]. It is apparent that these timing data are worthwhile data to collect and the interpretation of these data has important implications for patient safety practices. However, diverse data collection methods and approaches to analysis make comparisons across studies difficult and impede knowledge building in this field. This paper describes several challenges to quantifying the timing of prophylactic antibiotics that we encountered during a recent study (Lingard L, Regehr G, Cartmill C, Orser B, Espin S, Bohnen J, Reznick R, Baker R, Rotstein L, Doran D: Evaluation of a preoperative team briefing: Improved communication routine results in improved clinical practice, submitted) and offers a suggested protocol for resolving these challenges in order to standardize future analyses and facilitate the comparison of future studies in this field.

## Background

We implemented a pre-operative team briefing that used a checklist as a guide and included members from all three professions in the operating room: nursing, anesthesia and surgery. As a marker of the briefing's effect on patient safety, we evaluated its impact on the timing of antibiotic prophylaxis administration, using a pre-intervention, post-intervention study design. The original objective of this research was to analyze the antibiotic timing data as a binary variable (on-time, within one hour prior to incision or not-on-time) and perform a simple chi-squared analysis between the pre and post phases of the study. However, while the vast majority of studies that offer data on antibiotic prophylaxis timing present simple data collection methods that lead to straight-forward analyses and interpretation, we found that the collection of data on antibiotic prophylaxis timing was a complex process requiring intricate data deciphering, coding and decision-making about the specific data to include and exclude from analysis. Two clear challenges manifested during the data extraction process: the actual classification of antibiotic timing, and the additional complication of multiple antibiotic regimens with different timing classifications in a single case. Each of these will be elaborated in turn.

## Challenges

### Data extraction and classification

The first challenge was in attempting to fit all of the data extracted from the patient charts into one of two categories (on-time or not-on-time). For this study, antibiotics that were documented in the chart documents as administered within 1 hour prior to the time of incision recorded on the operative record were coded as *on-time*, a time period used extensively in the current literature to distinguish timely and untimely antibiotic administration[1,3,4,10-16,18,19,22,24,31-33]. Antibiotics that were documented as administered outside of this 1 hour window were coded as *discordant *(clearly not on time).

However, we encountered many instances of incomplete, ambiguous, and unclear documentation in the chart documents in a manner that appears to have been experienced by other researchers who have tried to document antibiotic timing[3,4,8,9,13,15,19,20]. However, despite these identified ambiguities and problems of documentation in the literature, methods of handling complex chart data are quite inconsistent across the studies and reported only sporadically. Several studies report the exclusion of cases without documentation of the antibiotic administered or the time administered[3,4,15]. Several other studies make note of the variability in antibiotic administration documentation practices[9,13], but do not provide details of how this variability was accounted for in analyses. A few studies note the problem of missing data[8,19], but do not provide specific details of how these missing data were interpreted or dealt with in analyses.

Because of the high level of ambiguity and undocumented cases in our research sample (20% of cases) we were hesitant to exclude these cases from the sample. Thus, we developed a formalized protocol for dealing with these cases (Table 1). Situations where preoperative antibiotics were ordered but not documented as given, or where antibiotics were neither ordered nor documented as given but were a requirement of the procedure, were given a code of *not-documented*. Antibiotics that were documented as given, but without clear chart documentation of the associated timing, were coded as *ambiguous-timing*.

**Table 1:** Description of antibiotic codes applied to data documented in patient charts

Codes applied to data	Description of coded data	N cases with code*
On-time	Antibiotic documented; time documented; time within1 hour prior to incision	449

Discordant	Antibiotic request documented; delivery time documented; time not within 1 hour prior to incision	93

Not-documented	Antibiotic request not documented and delivery not documented	95

Ambiguous-timing	Antibiotic request documented; delivery documented but not with associated time	42

*Total cases collapsed across pre-test and post-test study phases.

For the purposes of analysis (to determine the effect of the intervention on antibiotic timing) the data describing various problems with timing were combined in a variety of ways to represent three levels of timeliness problems: clearly not on time; clearly problematic; and potentially problematic (Table 2). Separate χ^2 ^analyses were performed for these three different levels of timeliness problems for the purposes of understanding the impact of the various categorizations on the conclusions.

**Table 2:** Codes and categories included in each χ^2 ^analysis:

	categories used in analyses	Codes included in categories of untimeliness	N cases in category*
	On time		449

Analysis 1	clearly-not-on-time	discordant	93

Analysis 2	clearly-problematic	discordant + not documented	188

Analysis 3	potentially-problematic	discordant + not documented + ambiguous timing	230

*Total cases collapsed across pre-test and post-test study phases.

### Multiple antibiotic regimens

The second significant challenge we encountered was in interpreting prophylaxis regimens that involved multiple antibiotics. Every possible combination of *not-documented*, *ambiguous-timing*, *not-on-time *and *on-time *codes was encountered for procedures with multiple antibiotic regimens.

Again, this predicament has been described sporadically in the literature and dealt with in a variety of ways. Some studies have avoided the predicament by studying procedures and populations that routinely require a single antibiotic for prophylaxis[7,9,12,14,23]. Several studies evaluate each drug separately and then assess the complete antibiotic course by combining the separate evaluations, with divergences from the guidelines for one drug leading to a final assessment of the total prophylactic course as discordant with the guidelines[5,17,24]. Hawn et al. analyze only the first antibiotic administered in the dataset[4].

In our study, we decided that each case should have a single code regarding antibiotic timeliness even if multiple antibiotics were to be administered. To accomplish this, each antibiotic relevant to the case was evaluated independently and given an antibiotic code according to the rules described in table 1. Then, to assess overall antibiotic timeliness of the case, these codes were combined in a hierarchical fashion and one of these codes chosen to represent the entire case as follows. Given that patient safety is unequivocally at risk when antibiotics are given either too early or too late, relative to the time of incision [21-29], and based on the approach described in the literature[5,17,24], we decided that a case would be coded as *on-time *only if every prescribed or administered antibiotic in the procedure was given on time. For cases with at least one *discordant *antibiotic, the case was coded as *discordant*, regardless of the codes for the other antibiotics. A code *of not-documented *took precedence over a code of *ambiguous-timing*.

## Conclusion & implications for research

Researchers who are tracking prophylactic antibiotic timing as an outcome measure should be aware that documentation of antibiotic timing in the patient chart is frequently incomplete and unclear, and these inconsistencies should be accounted for in analyses. While some studies appear to have avoided encountering these complexities by using data collection methods such as 'real-time' data collection [5,31] or collecting antibiotic timing data in the OR[11,14,15], such techniques are not always possible, leading to reliance on the documentation from the patient chart. Previous studies provide hints for how to manage these complexities, but we have proposed a systematic method for dealing with specific patterns encountered in the data. There are several advantages to the method we have developed. The use of this hierarchical coding model and performing three separate χ^2 ^analyses allowed us to keep data in the sample rather than excluding a large proportion of cases because of poor documentation practices. Although the *clearly-not-on-time *analysis is most conservative and provides the most certainty that timeliness is due to real administration practices rather than documentation practices, it provides the smallest sample size, as it requires the elimination of cases with ambiguous timing in the documentation and cases with no documentation regarding antibiotics. By contrast, the *potentially-problematic *coding provides a larger sample, but we are less confident that the data represent administration rather than documentation practices. This method allows the researcher to balance the issues of sample size and data-accuracy and to explore this balance from three different levels of certainty. Future researchers will need to decide whether this proposed plan is suitable to the data sources and consider tailoring the system to account for variability in antibiotic characteristics (it may be appropriate to classify timeliness using a cut-off point other than 1 hr for certain antibiotics with different biological properties). While this analysis classifies antibiotic administration practices strictly based on timing, overall appropriateness of the antibiotic choice was not considered. Future research might investigate how to classify clearly inappropriate cases of antibiotic administration, such as the administration of a medication that is contra-indicated due to patient allergy, in the context of this type of classification system. We propose that the general adoption of a systematic approach to analysis of this type of data will allow for cross-study comparisons and ensure that interpretation of results is on the basis of timing practices rather than documentation practices.

## Competing interests

The authors declare that they have no competing interests.

## Authors' contributions

CC contributed to data acquisition, analysis and interpretation of data, statistical analysis, drafting of the manuscript, and provided administrative, technical and material support. LL contributed to study design, data acquisition, analysis and interpretation of data, drafting of the manuscript, obtaining funding and study supervision. GR contributed to study design, analysis and interpretation of data, statistical analysis and drafting of the manuscript. SE contributed to study design, data acquisition, analysis and interpretation of data and critical revision of the manuscript. JB contributed to study design, analysis and interpretation of data and critical revision of the manuscript. RB contributed to study design, analysis and interpretation of data and critical revision of the manuscript. LR contributed to study design, analysis and interpretation of data and critical revision of the manuscript.

## Pre-publication history

The pre-publication history for this paper can be accessed here:

http://www.biomedcentral.com/1471-2288/9/43/prepub

## References

[B1] Galandiuk S Rao MK Heine MF Scherm MJ Polk HC Mutual reporting of process and outcomes enhances quality outcomes for colon and rectal resections Surgery 2004 136 4 833 841 10.1016/j.surg.2004.06.021 15467669

[B2] Hing WC Yeoh TT Yeoh SF Lin RTP Li SC An evaluation of antimicrobial prophylaxis in paediatric surgery and its financial implication J Clin Pharm Ther 2005 30 371 381 10.1111/j.1365-2710.2005.00659.x 15985051

[B3] Bratzler DW Houck PM Richards C Steele L Dellinger P Fry DE Wright C Ma A Carr K Red L Use of antimicrobial prophylaxis for major surgery Arch Surg 2005 140 174 182 10.1001/archsurg.140.2.174 15724000

[B4] Hawn MT Gray SH Vick CC Itani KM Bishop MJ Ordin DL Houston TK Timely administration of prophylactic antibiotics for major surgical procedures J Am Coll Surg 2006 203 6 803 811 10.1016/j.jamcollsurg.2006.08.010 17116547

[B5] Tourmousoglou CE Yiannakopoulou EC Kalapothaki V Bramis J St. Papadopoulos J Adherence to guidelines for antibiotic prophylaxis in general surgery: a critical appraisal J Antimicrob Chemother 2008 61 214 218 10.1093/jac/dkm406 17999981

[B6] VanDisseldorp J Slingenberg EJMH Matute A Delgado E Hak E Hoepelman IM Application of guidelines on preoperative antibiotic prophylaxis in Leon, Nicaragua Neth J Med 2006 64 11 411 416 17179571

[B7] Bedouch P Labarere J Chirpaz E Allenet B Lepape A Fourny M Pavese P Girardet P Merloz P Saragaglia D Compliance with guidelines on antibiotic prophylaxis in total hip replacement surgery: results of a retrospective study of 416 patients in a teaching hospital Infect Control Hosp Epidemiol 2004 25 4 302 307 10.1086/502396 15108727

[B8] Silver A Eichorn A Kral J Pickett G Barie P Pryor V Dearie MB Timeliness and use of antibiotic prophylaxis in selected inpatient surgical procedures Am J Surg 1996 171 548 552 10.1016/S0002-9610(96)00036-0 8678197

[B9] Thonse R Sreenivas M Sherman KP Timing of antibiotic prophylaxis in surgery for adult hip fracture Ann R Coll Surg Engl 2004 86 263 266 15239868 10.1308/147870804560 PMC1964227

[B10] Awad SS Fagan SP Bellows C Albo D Green-Rashad B Garza MDL Berger DH Bridging the communication gap in the operating room with medical team training Am J Surg 2005 190 770 774 10.1016/j.amjsurg.2005.07.018 16226956

[B11] Carles M Gindre S Aknouch N Goubaux B Mousnier A Raucoules-Aime M Improvement of surgical antibiotic prophylaxis: a prospective evaluation of personalized antibiotic kits J Hosp Infect 2006 62 372 375 10.1016/j.jhin.2005.09.004 16337311

[B12] Wax DB Beilin Y Levin M Chadha N Krol M Reich DL The effect of an interactive visual reminder in an anesthesia information management system on timeliness of prophylactic antibiotic administration Anesth Analg 2007 104 6 1462 1466 10.1213/01.ane.0000263043.56372.5f 17513642

[B13] McCahill LE Ahern JW Gruppi LA Limanek J Dion GA Sussman JA McCaffrey CB Leary DB Lesage MB Single RM Enhancing compliance with Medicare guidelines for surgical infection prevention: experience with a cross-disciplinary quality improvement team Arch Surg 2007 142 355 361 10.1001/archsurg.142.4.355 17438170

[B14] Rosenberg AD Wambold D Kraemer L Begley-Keyes M Zuckerman SL Singh N Cohen MM Bennett MV Ensuring appropriate timing of antimicrobial prophylaxis J Bone Joint Surg Am 2008 90 226 232 10.2106/JBJS.G.00297 18245579

[B15] O'Reilly M Talsma A VanRiper S Kheterpal S Burney R An anesthesia information system designed to provide physician-specific feedback improves timely administration of prophylactic antibiotics Anesth Analg 2006 103 4 908 912 10.1213/01.ane.0000237272.77090.a2 17000802

[B16] Webb ALB Flagg RL Fink AS Reducing surgical site infections through a multidisciplinary computerized process for preoperative prophylactic antibiotic administration Am J Surg 2006 192 663 668 10.1016/j.amjsurg.2006.08.014 17071203

[B17] VanKasteren MEE Mannien J Kullberg BJ DeBoer AS Nagelkerke NJ Ridderhof M Wille JC Gyssens IC Quality improvement of surgical prophylaxis in Dutch hospitals: evaluation of a multi-site intervention by time series analysis J Antimicrob Chemother 2005 56 1094 1102 10.1093/jac/dki374 16234334

[B18] Parker BM Henderson JM Vitagliano S Nair BG Petre J Maurer WG Roizen MF Weber M DeWitt L Beedlow J Six Sigma methodology can be used to improve adherence for antibiotic prophylaxis in patients undergoing noncardiac surgery Economics, Education and Policy 2007 104 1 140 146 10.1213/01.ane.0000250371.76725.2e 17179259

[B19] Quenon JL Eveillard M Vivien A Bourderont D Lepape A Lathelize M Jestin C Evaluation of current practices in surgical antimicrobial prophylaxis in primary total hip prosthesis – a multicentre survey in private and public French hospitals J Hosp Infect 2004 56 202 207 10.1016/j.jhin.2003.11.005 15003668

[B20] Bull AL Russo PL Friedman ND Bennett NJ Boardman CJ Richards MJ Compliance with surgical antibiotic prophylaxis – reporting from a statewide surveillance programme in Victoria, Australia J Hosp Infect 2006 63 140 147 10.1016/j.jhin.2006.01.018 16621135

[B21] Classen DC Evans RS Pestotnik SL Horn SD Menlove RL Burke JP The timing of prophylactic administration of antibiotics and the risk of surgical-wound infection N Engl J Med 1992 326 5 281 286 1728731 10.1056/NEJM199201303260501

[B22] Olsen MA Nepple JJ Riew KD Lenke LG Bridwell KH Mayfield J Fraser VJ Risk factors for surgical site infection following orthopaedic spinal operations J Bone Joint Surg Am 2008 90 62 69 10.2106/JBJS.F.01515 18171958

[B23] Garey KW Dao T Chen H Amrutkar P Kumar N Reiter M Gentry LO Timing of vancomycin prophylaxis for cardiac surgery patients and the risk of surgical site infections J antimicrob chemother 2006 58 645 650 10.1093/jac/dkl279 16807254

[B24] VanKasteren MEE Mannien J Ott A Kullberg BJ DeBoer AS Gyssens IC Antibiotic prophylaxis and the risk of surgical site infections following total hip arthroplasty: Timely administration is the most important factor Clin Infect Dis 2007 44 921 927 10.1086/512192 17342642

[B25] Bernard HR Cole WR The prophylaxis of surgical infection: the effect of prophylactic antimicrobial drugs on the incidence of infection following potentially contaminated operations Surgery 1964 56 151 157 14174732

[B26] Burke JP Maximizing appropriate antibiotic prophylaxis for surgical patients: an update from LDS Hospital, Salt Lake City Clin Infect Dis 2001 33 suppl 2 S78 S83 10.1086/321861 11486303

[B27] Martin C Antimicrobial prophylaxis in surgery: general concepts and clinical guidelines French Study Group on Antimicrobial Prophylaxis in Surgery, French Society of Anesthesia and Intensive Care Infect Control Hosp Epidemiol 1994 15 7 463 471 7963438 10.1086/646952

[B28] Stone HH Hooper A Kolb LD Geheber CE Dawkins EJ Antibiotic prophylaxis in gastric, biliary and colonic surgery Ann Surg 1976 184 4 443 452 827989 10.1097/00000658-197610000-00007 PMC1345439

[B29] Bratzler DW Houck PM For the Surgical Infection Prevention Guideline Writers Workgroup. Antimicrobial prophylaxis for surgery: an advisory statement from the National Surgical Infection Prevention Project Am J Surg 2005 189 395 404 10.1016/j.amjsurg.2005.01.015 15820449

[B30] Burke JF The effective period of preventive antibiotic action in experimental incisions and dermal lesions Surgery 1961 50 161 168 16722001

[B31] Altpeter T Luckhardt K Lewis JN Harken AH Polk HC Expanded surgical time out: a key to real-time data collection and quality improvement J Am Coll Surg 2007 204 4 527 532 10.1016/j.jamcollsurg.2007.01.009 17382210

[B32] Peipert JF Weitzen S Cruickshank C Story E Ethridge D Lapane K Risk factors for febrile morbidity after hysterectomy Obstet Gynecol 2004 103 1 86 91 14704250 10.1097/01.AOG.0000109219.24211.30

[B33] White A Schneider T Improving compliance with prophylactic antibiotic administration guidelines AORN J 2007 85 1 173 180 10.1016/S0001-2092(07)60023-4 17223406

